# Beyond Sterilization: A Comprehensive Review on the Safety and Efficacy of Opportunistic Salpingectomy as a Preventative Strategy for Ovarian Cancer

**DOI:** 10.3390/curroncol30120739

**Published:** 2023-11-28

**Authors:** Tahereh Zadabedini Masouleh, Holly Etchegary, Kathleen Hodgkinson, Brenda J. Wilson, Lesa Dawson

**Affiliations:** 1 Clinical Epidemiology Program, Faculty of Medicine, Memorial University of Newfoundland, St. John’s, NL A1B 3V6, Canada; tzadabedinim@mun.ca; 2 Division of Community Health and Humanities, Faculty of Medicine, Memorial University, St. John’s, NL A1B 3V6, Canada; 3 Division of Community Health and Humanities, Memorial University of Newfoundland, St. John’s, NL A1B 3V6, Canada; 4 Division of Biomedical Sciences, Memorial University of Newfoundland, St. John’s, NL A1B 3V6, Canada; 5 Discipline of Obstetrics and Gynecology, Faculty of Medicine, Memorial University, St. John’s, NL A1B 3V6, Canada

**Keywords:** opportunistic salpingectomy, prophylactic salpingectomy, ovarian cancer, prevention, surgical complication, ovarian reserve, risk, safety, efficacy

## Abstract

Ovarian cancer (OC) is Canada’s third most common gynecological cancer, with an estimated 3000 new cases and 1950 deaths projected in 2022. No effective screening has been found to identify OC, especially the most common subtype, high-grade serous carcinoma (HGSC), at an earlier, curable stage. In patients with hereditary predispositions such as *BRCA* mutations, the rates of HGSC are significantly elevated, leading to the use of risk-reducing salpingo-oophorectomy as the key preventative intervention. Although surgery has been shown to prevent HGSC in high-risk women, the associated premature menopause has adverse long-term sequelae and mortality due to non-cancer causes. The fact that 75% of HGSCs are sporadic means that most women diagnosed with HGSC will not have had the option to avail of either screening or prevention. Recent research suggests that the fimbrial distal fallopian tube is the most likely origin of HGSC. This has led to the development of a prevention plan for the general population: opportunistic salpingectomy, the removal of both fallopian tubes. This article aims to compile and review the studies evaluating the effect of opportunistic salpingectomy on surgical-related complications, ovarian reserve, cost, and OC incidence when performed along with hysterectomy or instead of tubal ligation in the general population.

## 1. Introduction

Ovarian cancer (OC) is the most lethal gynecological cancer with the worst prognosis [1]. According to the American Cancer Society, the lifetime risk of developing OC is 1 in 78, and it is fatal in 1 out of 108 women [2], with a median age of 63 at diagnosis and 70 at death [3]. Age, family history, endometriosis, obesity, hormone replacement therapy, and a greater height are risk factors for OC [1,4]. In contrast, oral contraceptive use and a higher number of pregnancies have been shown to have a protective effect [5]. OC is a significant public health concern, with a high mortality rate. Sung et al. reported 313,959 new cases and 207,252 deaths in 2020 globally, with a mortality rate of 66%. North America has the third highest incidence rate, with 26,630 cases, behind Asia and Europe [6]. The projected incidence rate of OC in Canada for 2022 is 3000 cases, with 1950 projected deaths [7].

OC presents a significant challenge due to late-stage diagnoses and non-specific symptoms, resulting in a low survival rate. The overall 5-year relative survival rate for OC is merely 49.7%, with minimal improvement over the years [3]. In the early stages of the disease (I and II), in which only 15 to 19% of cases are diagnosed, the 5-year survival rate is between 70% and 90%, drastically decreasing to 17% in stage IV [8]. Additionally, tumor cell type plays a role in survival rates, with borderline OC showing the best prognosis and epithelial OC demonstrating the worst survival rate among all OC types [8]. Epithelial OC is the most common subtype, accounting for up to 95% of malignant cases, with high-grade serous carcinoma (HGSC) being the predominant histotype, accounting for over 70% of epithelial OC cases [9]. HGSC is characterized by ubiquitous somatic TP53 mutations, leading to high invasiveness and a poor prognosis [10]. About 15% of all OCs [11] and 25% of all HGSCs [12] are hereditary, often linked to *BRCA*1/2 gene mutations [13]. However, most OC cases occur sporadically and have worse survival and prognosis than familial cases [13,14,15]. Despite the need for effective screening methods, two large RCTs in the UK and the US did not find significant improvements in survival rates after intervening early screening, highlighting the necessity of a preventative strategy in the general population [16,17].

Understanding the origin of OC is vital for prognosis and prevention. Previous theories implicated the ovarian surface epithelium (OSE) but failed to explain diverse histotypes and genomic profiles [18,19]. Recent evidence suggests that the distal fallopian tube may be the origin of HGSCs [20]. Studies have identified dysplastic and hyperplastic changes in the fallopian tube fimbriae of women with *BRCA* mutations, known as serous tubal intraepithelial carcinomas (STICs), which share features with HGSC [21]. Utilizing a protocol called sectioning and extensively examining the fimbrial end of the fallopian tube (SEE-FIM) has led to the detection of precursor lesions in HGSC in both high- and low-risk populations [22]. These findings indicate that the fallopian tubes are likely the primary site of origin for most serous ovarian carcinomas, and, therefore, opportunistic salpingectomy (OS) may hold promise for HGSC prevention in the general population in addition to the high-risk population.

## 2. What Is Opportunistic Salpingectomy?

Salpingectomy involves the removal of one or both fallopian tubes surgically, typically for contraception or the treatment of fallopian tube abnormalities, such as ectopic pregnancy or hydrosalpinx, whereas opportunistic, risk-reducing, or prophylactic salpingectomy refers to the removal of both normal fallopian tubes during pelvic surgeries while preserving the ovaries [23].

In September 2010, the gynecologic cancer research team OVCARE in BC urged gynecologists to consider performing bilateral salpingectomy at the time of hysterectomy and as an alternative to tubal ligation when women at population risk seek permanent contraception [24]. Their study demonstrated a significant increase in the rate of hysterectomy with bilateral opportunistic salpingectomy (BOS) from 5% (2008) to 35% (2011) of all hysterectomy procedures in BC, Canada, with most of this change occurring after September 2010. Additionally, the number of bilateral salpingectomies for sterilization in place of tubal ligation increased by 22% in one year [25]. In 2011, the Society of Obstetricians and Gynecologists of Canada recommended that physicians consider the practice of salpingectomy during benign gynecologic surgeries in the general population when childbearing is complete [26]. As a result, in Canada (excluding the province of Quebec), the rate of hysterectomy with BOS increased by 20% from 2011 to 2016 [27], indicating an increasing trend in the adoption of salpingectomy in gynecologic surgeries in the country.

## 3. Opportunistic Bilateral Salpingectomy during Hysterectomy

Hysterectomy ranks as the second most frequent surgical procedure in women after cesarean section [28], and its prevalence is influenced by factors like age, ethnicity [29], race [30], and socioeconomic status [31]. In total, 90% of the hysterectomies performed are due to benign diseases, mainly uterine fibroids, abnormal uterine bleeding, and endometriosis, totaling around 400,000 inpatient procedures annually in the US [32]. Canada has a similarly high rate of hysterectomy, with about one-third of women undergoing the procedure by age 60 [33]. The age-standardized rate for this surgery was 234 per 100,000 cases in 2021 in Canada (excluding the province of Quebec), with Saskatchewan recording the highest rate at 326 per 100,000 [34].

In recent years, surgical techniques have evolved, favoring minimally invasive approaches like laparoscopic and robotic-assisted hysterectomy for benign reasons [35]. This shift has led to increased outpatient procedures and same-day discharges due to reduced complications, lowered medical costs [36], and improved feasibility [37,38]. Notably, Moawad et al. showed that 44% of hysterectomies for benign indications shifted to same-day discharge between 2008 and 2014 [39]. It is estimated that approximately 100,000 to 200,000 outpatient hysterectomies are carried out annually in the US [40]. Given the large number of hysterectomies performed each year, the incorporation of bilateral salpingectomy creates an opportunity to remarkably increase the adoption of this procedure among premenopausal women and potentially reduce OC incidence on a substantial scale. However, this approach also raises important considerations regarding safety, effects on ovarian function, and cost-effectiveness, which is thoroughly explored in the following section.

### 3.1. Surgical and Post-Surgical Complications of Hysterectomy with Salpingectomy Regardless of the Approach

Hysterectomy can be performed in different settings and with differing surgical approaches, laparotomy, and vaginal or minimally invasive techniques. Several studies have evaluated the surgical complications associated with concomitant salpingectomy while considering all approaches combined. The main objective measures of the surgical complications assessed in these studies include the length of hospitalization and operation, blood transfusion and readmission rates, and estimated blood loss (EBL). In the following section, a summary of these studies is presented.

Three retrospective studies examined peri- and postoperative complications and found no significant increase in adverse events when salpingectomy was added to hysterectomy [41,42,43]. A nationwide Canadian registry-based study comparing 10,697 cases with bilateral salpingectomy to 195,238 cases with hysterectomy alone showed no differences in blood transfusion, hospital stay, post-surgical fever, or infection [41]. Similarly, no significant changes in EBL, the length of stay, or the occurrence of any events causing complications during or after the surgery were reported by a retrospective cross-sectional study, including 4890 cases with OBS [42]. A multicenter clinical trial also supported these findings, showing no increase in operative time, blood loss, complications, or hospitalization with the addition of bilateral salpingectomy to hysterectomy [43].

Regarding surgery duration, two studies indicated a modest increase when bilateral salpingectomy was added to hysterectomy. Till et al. reported an average 12 min increase in operation time regardless of surgical approach [42]. This is supported by another population-based cohort study in the province of BC, Canada (2008–2011), which indicated an average 16 min extension of operation time [25]. Of interest, the hospitalization duration was shorter by an average of 3.6 h in those who had bilateral salpingectomy. Other than that, no statistically significant differences were observed regarding the readmission and blood transfusion rates in both groups [25]. These findings align with the result of another cohort study in which only laparoscopic and abdominal approaches were included. No significant differences in surgical or post-surgical-related complications between both groups were shown, except for a 10.2 h reduction in hospitalization for the OBS group, the mean length of hospitalization [44]. In contrast, a separate retrospective cohort study comparing laparoscopic or abdominal hysterectomy with or without salpingectomy reported longer hospitalization by 2 h and 24 min in the salpingectomy group [95% CI 0.02–0.18] but with 20 mL less blood loss [95% CI 0.02–0.18] [45].

A retrospective cohort study evaluating minor postoperative complications reported that performing salpingectomy with hysterectomy, regardless of approach, did not increase the rate of physician visits for any surgery-related complications or infections two weeks after being discharged. The only increased risk for the OBS group was a 20% higher likelihood of being prescribed analgesics during those two weeks, which disappeared after one month [46].

Overall, the evidence evaluating all types of hysterectomy, regardless of approach, suggests that the addition of salpingectomy to any route of hysterectomy appears safe and does not increase complications, apart from a modest increase in the duration of surgery. Although the findings on hospitalization duration are mixed, most studies did not show the negative effects of salpingectomy on this parameter. Further research is encouraged to better understand the benefits and potential risks associated with incorporating bilateral salpingectomy during hysterectomy. 

### 3.2. Ovarian Reserve

The fallopian tubes run alongside the ovary, raising concerns about the potential compromise of blood supply to the ovaries and subsequent impact on ovarian reserve or early menopause due to salpingectomy. Premature surgical menopause is associated with multiple negative long-term sequelae, such as early osteoporosis, cardiac disease, and dementia, making the long-term safety of salpingectomy a crucial consideration.

To understand the effect of salpingectomy on ovarian reserve, a meta-analysis included eight studies with a follow-up time of 3 to 18 months in which cases the fallopian tubes were removed either through laparoscopic hysterectomy, through sterilization, or due to ectopic pregnancy. The pooled results showed no significant changes in anti-Müllerian hormone (AMH) serum levels after salpingectomy, suggesting no short-term negative impact on ovarian reserve [47].

However, a prospective study on 84 women who underwent hysterectomy with bilateral salpingectomy reported a significant decline in AMH levels (delta AMH = −0.49 ng/mL *p* < 0.001) and a significantly higher level of follicle-stimulating hormone (FSH) (delta FSH = −7.21 mIU/mL *p* < 0.001) six weeks postoperatively, suggesting diminished ovarian reserve after hysterectomy with bilateral salpingectomy [48]. It is worth noting that this study had a relatively short follow-up period, which could have influenced the hormonal levels since they tend to be unstable after adnexal surgery [49]. Moreover, 37% (31/84) of patients had cervical cancer, which has been shown to lower the ovarian reserve and can be a confounder in the analysis [50]. The reported extent of FSH change was relatively small, and some authors would argue that this level of difference is not clinically significant or a meaningful predictor of true increased rates of menopause.

A clinical trial examining the levels of FSH and luteinizing hormone (LH) before and six months after hysterectomy with/without salpingectomy revealed elevated levels of both hormones at six months postoperatively in both groups, with no significant differences between the groups, indicating no increased risk of impaired ovarian function due to salpingectomy [51]. A prospective cohort study of 859 patients who completed a follow-up at 48 months in which FSH, LH, and estradiol (E2) levels and perimenopausal symptoms were checked showed no significant hormonal level difference at the 48th month other than a lower level of FSH in the salpingectomy group (34.9 U/L) than in the hysterectomy-only group (38 U/L; *p* = 0.043). However, at 24 months, the number of patients experiencing perimenopausal symptoms was 7.3% higher in the no-salpingectomy group, and the salpingectomy group had a significantly lower rate of pelvic pseudocysts [52].

Measurements of the AMH concentration before and six months after surgery in a clinical trial, including abdominal or laparoscopic hysterectomies, demonstrated that the addition of bilateral salpingectomy does not significantly alter ovarian reserve [43]. Likewise, a prospective study comparing AMH and FSH levels three months after surgery in women who underwent hysterectomy with or without OBS found no significant differences either within or between groups [53].

In conclusion, the available evidence suggests that salpingectomy during hysterectomy does not adversely affect ovarian reserve. However, further research with longer follow-up periods is essential to confidently assess the impact of salpingectomy on ovarian function and its overall safety during hysterectomy procedures.

## 4. Total Salpingectomy instead of Tubal Ligation

In 2019, approximately 12% of women worldwide had undergone a form of permanent sterilization, making it the most common form of contraception [54]. Supporting evidence on the preventative role of OBS has shifted the purpose of this surgery from treatment for certain medical conditions, such as ectopic pregnancies or the presence of hydrosalpinx, to a contraception method [55].

The uptake of postpartum and interval opportunistic salpingectomy as a mode of sterilization is increasing. A multicenter cohort study demonstrated an approximately 72% increase in the interval salpingectomy rate between 2013 and 2016, with an opposite trend in the rate of bilateral tubal ligation over the study period [56]. Wagar et al. showed that 80% of all postpartum sterilizations after vaginal delivery occurred through salpingectomies in 2019, compared to 5.9% in 2014 [57]. 

### 4.1. Surgical and Post-Surgical Complications of Salpingectomy instead of Tubal Ligation

When comparing bilateral salpingectomy with tubal ligation (TL), McAlpine et al. reported an increased length of operation by an average of 10 min in those who underwent salpingectomy for sterilization (61 min in the TL group vs. 71.2 min in the OS group; *p* < 0.001), but no significant differences were observed for the length of hospital stay, rate of readmission, or blood loss [25].

A meta-analysis performed on five RCTs compared surgical-related complications, including the duration of operation and hospitalization, blood loss, changes in hemoglobin, the risk of wound infections, rehospitalization, reoperation, and other postoperative complications in bilateral salpingectomy vs. tubal ligation. The results showed no significant difference in the aforementioned parameters between the two groups [58].

Many patients request sterilization in the immediate postpartum period or at the time of cesarean section. Salpingectomy can therefore be performed in three circumstances: during cesarean delivery, within 24 to 48 h after vaginal delivery, or as a non-postpartum interval procedure. In the following, the surgical-related complications of each scenario vs. tubal ligation are reviewed.

The majority of studies focused on salpingectomy during cesarean delivery. A meta-analysis, including nine observational and experimental studies, reported six minutes of extra operative time in the salpingectomy group during cesarean delivery compared to tubal ligation, while no significant difference with regard to intra- or postoperative complications was observed between the two groups [59]. The same results were obtained by an additional meta-analysis on 11 studies in which the only significant difference was a 6.3 min longer operative time in eight cohort studies [60]. A more recent retrospective cohort study also reported comparable results when comparing tubal occlusion with total salpingectomy at the time of cesarean delivery, with a 6.5 min difference in operative time in favor of tubal occlusion [61].

With bilateral salpingectomy as a non-postpartum interval procedure, a retrospective cohort study assessed its feasibility and safety compared with laparoscopic tubal ligation. Both groups showed comparable intra- and postoperative complications, except for the average operative time, which was 11 min longer in the laparoscopic salpingectomy group (*p* < 0.0001) [56]. The findings from another cohort study also showed no significant changes in EBL or complications when interval salpingectomy was performed instead of tubal ligation. The operation time was reported to be 6 min longer in laparoscopic salpingectomy, but it was not statistically significant [62].

The available evidence suggests that bilateral salpingectomy after vaginal delivery does not substantially increase the rate of complications. A single-centered retrospective case series studied postpartum sterilization after vaginal delivery and found that the average surgical time was 11.31 min longer in the bilateral salpingectomy cohort via mini-laparotomy (*p* = 0.003) vs. tubal ligation using Pomeroy or Parkland techniques, but there were no significant differences in EBL or complication rates [63]. However, the results of a cohort study showed that bilateral salpingectomy operation on women who have delivered vaginally takes 4 min less and has slightly more EBL (5 mL) than bilateral tubal ligation (*p* = 0.03 and 0.15, respectively). Other examined parameters, including the length of hospitalization, the risk of readmission, and emergency visits, were similar between the two groups [64]. The shorter operative time and lower amount of blood loss in the salpingectomy group in the mentioned study may be due to the fact that 94% (106/113) of all bilateral salpingectomies were performed using a bipolar electrocautery device [64].

A retrospective cohort study consisted of two sets of comparisons, namely, one for salpingectomy after vaginal delivery and one for salpingectomy with cesarean delivery, and it showed that, in both groups, salpingectomy had a statically significant but modestly longer operation time than tubal ligation (the addition of 10 and 9.9 min, *p* = 0.05, respectively), whereas similar rates of blood loss were stated for salpingectomy in both types of deliveries vs. tubal ligation [65].

Overall, when comparing bilateral salpingectomy with tubal ligation for sterilization, there are data reporting that bilateral salpingectomy may result in longer operation times. However, this difference is not statistically significant in all studies. There is also no significant difference in the length of hospital stay, rate of readmission, or blood loss between the two groups. However, when considering the specific circumstances of the surgery, such as whether it is performed during cesarean delivery or as an interval procedure, there may be slight differences in operation time and blood loss. These inconsistent findings are likely attributed to the heterogeneity of surgical techniques and study designs. In conclusion, the evidence suggests that bilateral salpingectomy is a feasible and safe alternative to tubal ligation, with similar rates of complications.

### 4.2. Ovarian Reserve

Multiple studies have examined the effect of bilateral salpingectomy as an alternative to tubal ligation on ovarian reserve, focusing on evaluating hormonal and ultrasonographic markers. A triad-center clinical trial compared the effect of bilateral salpingectomy with a bipolar device and bilateral partial salpingectomy on ovarian reserve in women undergoing cesarean delivery after one year. The results showed no significant differences between the two procedures in terms of hormonal (AMH and FSH) and ultrasonographic (PSV, AFC, VI, FI, ovarian volume, and calculated ovarian age) parameters [66]. In another randomized trial, the measurement of AMH levels before and six–eight weeks postdelivery in women who underwent salpingectomy via monopolar electrosurgery or tubal ligation using the Parkland method during C-section showed no significant difference either within or between groups [67]. Similarly, a prospective cohort study showed no statistically significant differences in AMH, FSH, or E2 levels between laparoscopic tubal ligation, bipolar bilateral salpingectomy, and healthy controls at one month or three months after surgery [68]. Pooled data from five studies in a recent meta-analysis showed no significant difference in FSH hormone levels between salpingectomy and proximal tubal ligation cohorts [69]. With regard to antral follicle count (AFC) and AMH, those with bilateral salpingectomy had higher levels in the short term (AFC: mean difference −0.80 IU/L, 95% CI [−1.46, −0.14]; AMH: mean difference −1.01 IU/L, 95% CI [−1.28, −0.74]) [69]. However, a subsequent prospective study compared AMH levels and AFC three and six months following cesarean delivery with bilateral salpingectomy with those who only had a C-section, with no significant changes in either marker reported at any time point between the study groups [70]. In summary, the results of these studies suggest that bilateral salpingectomy is not associated with a significant difference in ovarian reserve compared to tubal ligation as measured by hormonal and ultrasonographic parameters in the short term.

## 5. Cost-Effectiveness

OC imposes a significant economic burden on individuals, the healthcare system, and society as a whole [71,72,73]. Moreover, studies show that the families of OC patients also shoulder the economic impact, as they allocate time and/or resources to caregiving [74,75]. OC is one of the highest-cost cancers similar to brain, esophageal, and gastric cancers [76], and it has the highest healthcare cost per patient amongst gynecologic cancers in the US [77]. Diagnosis at an advanced stage of this cancer is associated with early progression (within 12 months) of the disease and, therefore, a higher level of financial costs [78]. A US study of 2991 cancer patients with private insurance who were <65 years old showed that their all-cause total cost was annually USD 104,964 more than the respective control cohort [79]. This aligns with the USD 93,632 expenditure reported on the care of commercially insured women with OC during the first year after surgery [80]. To assess the average cost of treatment for older patients, Urban et al. focused on Medicare users with late-stage OC, for whom it was estimated to be USD 65,908 for the first year following diagnosis [81]. Frailty is also shown to be associated with a greater cost of care in OC patients [82]. The evidence reviewed here highlights the need for cost-saving approaches to lighten the financial burden on society.

A number of studies have investigated the socioeconomic aspect of opportunistic salpingectomy. Kwon et al. studied the cost-effectiveness of opportunistic salpingectomy in the Canadian healthcare system for the first time based on life expectancy gain in a decision model analysis in which they found that opportunistic salpingectomy is a cost-effective approach compared to hysterectomy with or without bilateral salpingo-oophorectomy and instead of tubal ligation and that it can also be cost-saving in the long term [83]. These findings are supported by Dilley et al.’s study in which opportunistic salpingectomy was shown to be cost-effective based on gained quality-adjusted life years assuming a minimum prevention rate of 54% for OC using data from the US [84]. Their model also predicted that bilateral salpingectomy is a cost-saving option when performed with laparoscopic hysterectomy [84]. A decision analysis, with a focus on vaginal hysterectomy as a more complex surgical approach, showed that the addition of bilateral salpingectomy to the operation increases major complications by 0.61 for every cancer case prevented and is cost-effective with or without the inclusion of the cancer treatment costs [85]. In a conservative model, the mortality rate caused by OC was reduced by 8.13% and 6.34% when opportunistic salpingectomy was compared with tubal ligation and when hysterectomy with opportunistic salpingectomy was compared with hysterectomy alone, respectively, which leads to savings of USD 445 million per year in the US [86]. Including a wider number of laparoscopic non-gynecologic procedures along with hysterectomy and tubal ligation, such as appendectomy, colon resection, hernia, and cholecystectomy, in an analysis model demonstrated favorable results to the addition of opportunistic salpingectomy, along with the opportunity to save approximately USD 877M in the US annually [87,88].

For postpartum sterilization, two studies investigated the socioeconomic benefits of opportunistic salpingectomy solely at the time of cesarean delivery [89,90]. Both models identified opportunistic salpingectomy as a cost-effective alternative to tubal ligation when particular conditions are met. Venkatesh et al. defined a minimum 52% risk reduction and no more than 2% perioperative morbidity compared with tubal ligation for salpingectomy [89]. In contrast, the minimum risk reduction in Subramaniam’s model was 41% with a cost difference of USD 3163.74 between opportunistic salpingectomy and tubal ligation [91]. These results seem promising with the advent of novel low-cost approaches to salpingectomy at the time of C-section [90]. In a recent decision analysis study, Wager et al. evaluated the cost-effectiveness of opportunistic salpingectomy following vaginal delivery and estimated that there would be USD 6.48 million in cost savings when chosen over tubal ligation [92]. In regard to different forms of sterilization, the economic impacts of laparoscopic tubal ligation, tubal clips, and laparoscopic bilateral salpingectomy were compared by Tai et al., and bilateral salpingectomy was introduced as the most cost-effective strategy for sterilization [93]. The simulation model, including 10,000 women, showed that bilateral salpingectomy might reduce healthcare expenditure by USD 7823 and USD 6325 per life year gained compared to tubal clips and tubal ligation, respectively [93]. The cost-effectiveness of OBS is still being studied and is currently a topic of ongoing research, especially due to the lack of population-based data; however, based on the theoretical decision model, it appears to be cost-effective and cost-saving under some circumstances.

## 6. Efficacy of Opportunistic Bilateral Salpingectomy

In recent years, the implementation of OBS as a strategy for reducing the risk of OC has gained attention in the medical community. Several studies have been conducted to evaluate the effectiveness of this intervention on the incidence of OC in the general population. In this section, we aim to review the findings from six studies that focus on the topic of opportunistic salpingectomy and its impact on reducing the risk of OC. A comprehensive summary of the articles reviewed can be found in Table 1.

A nationwide case–control study conducted in Denmark between 1982 and 2011 found that bilateral salpingectomy is associated with a 42% decrease in the incidence of epithelial ovarian cancer (EOC) [94]. A retrospective Swedish population-based cohort study conducted between 1973 and 2009 observed a 35% lower risk of OC in the salpingectomy group vs. the control group after an average of 18 years of follow-up [95]. Additionally, a sub-analysis comparing the effects of unilateral with bilateral salpingectomy showed that bilateral salpingectomy was associated with an additional 50% decrease in the risk of OC compared to unilateral salpingectomy (unilateral salpingectomy: HR = 0.71 95% CI = 0.56–0.91; bilateral salpingectomy: HR = 0.35 95% CI = 0.17–0.73) [95]. A US-based case–control study also reported that excisional tubal sterilization, including complete and partial salpingectomy and distal fimbriectomy, was associated with a 64% reduced risk of EOC and primary peritoneal cancer (PPC) compared to controls without sterilization or with non-excisional tubal sterilization [96]. A meta-analysis of the aforementioned three studies found a 49% decrease in the incidence rate of OC after bilateral salpingectomy (OR = 0.51, 95% CI = 0.35–0.75, I2 = 0%) [97].

In their single-center case–control study, Chen et al. found that salpingectomy for benign reasons can decrease the overall EOC rate by approximately 52% compared to women whose fallopian tubes had been reserved [98]. Moreover, a retrospective case–control study with the aim of assessing the effects of hysterectomy, salpingectomy, and tubal ligation on the risk of EOC Types I and II was carried out while including cases diagnosed with EOC or PPC from 2008 to 2014 in Sweden. The findings specific to salpingectomy suggest that this surgical procedure was linked with a significant reduction in the risk of EOC Type II (Type II consists of HGSC, undifferentiated carcinoma, and malignant mixed mesodermal carcinomas), with a risk reduction of 38% [99]. 

**Table 1:** Characteristics of six observational articles included in this review.

Article	Madsen et al. [94]	Falconer et al. [95]	Lessard et al. [96]	Chen et al. [98]	Darelius et al. [99]	Hanley et al. [100]
**Design**	Nationwide case–control	Retrospective cohort	Population-based nested case–control	Case–control	Nationwide case–control	Retrospective cohort
**Country**	Denmark	Sweden	USA	China	Sweden	Canada (BC)
**Study period**	1982–2011	1973–2009	1966–2009	2007–2017	2008–2014	2008–2017
**Cases**	Patients with EOC n = 13,241	Previous gynecologic surgery on benign indications n = 251,465	All patients with EOC or PPC n = 194	Patients with EOC or PPC and history of gynecological surgery for benign reason n = 198	Patients with EOC, fallopian tube cancer, or PPC n = 4040	Previous hysterectomy with OS n = 14,066, or OS for sterilization n = 11,823
**Controls**	15 age-matched controls per case n = 194,689	Unexposed women (no surgery) n = 5,449,119	2 age-matched controls per case n = 388	2 age-matched controls with no previous OC n = 389	10 age-matched controls n = 39,100	Previous hysterectomy (alone) n = 10,446, or tubal ligation n = 21,634
**Exclusion**	Previous cancer, previous bilateral oophorectomy (controls only)	Primary OC and/or any gynecologic surgery before entering the cohort, inconsistencies in the data, emigration out of Sweden	Not residing in Olmsted County, previous fallopian tube carcinoma, non-serous cancers	Previous OC	Unable to subtype, previous EOC, previous bilateral oophorectomy	Any previous gynecological cancer
**Outcome**	EOC and borderline ovarian tumors	Ovarian and tubal cancer	Serous EOC or PPC	EOC or PPC	Types I and II EOC	Serous and epithelial OC
Confounder *	Age, parity, tubal ligation	Age, parity, calendar year, education status	Hysterectomy, salpingo-oophorectomy, use of oral contraceptive, endometriosis, parity, gravidity	Age, child number, menopause status	Pelvic inflammatory disease, endometriosis, other surgical procedures	NA
Result **	Unilateral salpingectomy: OR: 0.90 95% CI: 0.72–1.12 Bilateral salpingectomy: OR: 0.58 95% CI: 0.36–0.95	HR: 0.65 95%CI: 0.52–0.81 *p* = 0.0001	OR: 0.36 95%CI: 0.13–1.02 *p* = 0.054	OR: 2.080 95%CI: 1.340–3.227 *p* = 0.001	Type I: OR: 1.16 95%CI: 0.75–1.78 *p* = 0.51 Type II: OR: 0.62 95%CI: 0.45–0.85 *p* = 0.0032	Number of observed vs. expected serous cancer and EOC were 0 vs. 5.27 and 5 or less vs. 8.68 cases, respectively

* The confounders included in the fully adjusted model are named. ** Only the results specific to salpingectomy and from a fully adjusted model are displayed.

These findings are supported by the most recent retrospective cohort study conducted by Hanley et al. in the province of British Columbia, Canada. The study findings show that the observed rates of EOC and serous OC in the OS group, including 25,889 individuals, and in the control group, including 32,080 individuals who had hysterectomy alone or tubal ligation, were <=5 vs. 21 and 0 vs. 15, respectively [100]. Importantly, the calculated expected case numbers, based on the age-adjusted incident rate in the control group and follow-up duration, were 8.68 (for EOS) and 5.27 (for serous OC), which were greater than the observed rates of less than or equal to 5 (for EOS) and 0 (for serous OC) in the OS group [100]. Due to the relatively recent implementation of this preventative strategy and the long latency period of OC, we have only retrospective studies to inform evidence, which together suggest a 35–65% risk reduction in OC in the general population after salpingectomy.

## 7. Conclusions

In conclusion, the available data suggest that opportunistic bilateral salpingectomy during pelvic surgeries or as tubal sterilization is a safe procedure with minimal complications. The addition of bilateral salpingectomy to hysterectomy appears to be a viable option with minimal added risk of surgical and post-surgical complications, regardless of the surgical approach. The comparison between bilateral salpingectomy and tubal ligation for sterilization reveals several important findings. Bilateral salpingectomy has emerged as a viable alternative to tubal ligation, with a shift in its purpose from treatment for specific medical conditions to a method of contraception. The procedure shows comparable rates of complications to tubal ligation, with minor differences in operation time and blood loss depending on specific circumstances and surgical techniques.

Overall, opportunistic salpingectomy has emerged as a promising strategy for reducing the risk of OC. Although a longer follow-up time and prospective studies will be required to strengthen the evidence, the existing retrospective studies have demonstrated a significant decrease in OC incidence following bilateral salpingectomy, with risk reductions ranging from 35% to 65% in the general population. 

Opportunistic salpingectomy holds promise in reducing the risk of OC and can be safely implemented in most OB-GYN practices. Ongoing research and long-term follow-up studies are essential to fully understand its impact on OC incidence and optimize its implementation in clinical practice.
